# Livestock-associated methicillin and multidrug resistant *S. aureus* in humans is associated with occupational pig contact, not pet contact

**DOI:** 10.1038/srep19184

**Published:** 2016-01-12

**Authors:** Xiaohua Ye, Yanping Fan, Xiaolin Wang, Weidong Liu, Haifeng Yu, Junli Zhou, Sidong Chen, Zhenjiang Yao

**Affiliations:** 1 School of Public Health, Guangdong Pharmaceutical University, Guangzhou, China; 2 Leliu Hospital of Shunde, Shunde, China

## Abstract

This study aimed to explore the association of livestock-associated *S. aureus* with occupational pig contact and pet contact. In this cross-sectional study, 1,422 participants (including 244 pig workers, 200 pet-owning workers and 978 control workers) responded to a questionnaire and provided a nasal swab for *S. aureus* analysis. Resulting isolates were tested for antibiotic susceptibility, the immune evasion cluster (IEC) genes, and multilocus sequence type. Compared with controls, the pig workers demonstrated a greater prevalence of multidrug-resistant *S. aureus* (MDRSA) [prevalence ratio (PR) = 3.38; 95% CI: 2.07–5.53] and methicillin-resistant *S. aureus* (MRSA) (PR = 7.42; 95% CI: 3.71–14.83), but the prevalence of MDRSA and MRSA was similar in pet-owning workers and controls. There was a positive relation of frequency of pig contact with prevalence of MDRSA and MRSA carriage. Only pig workers carried MDRSA CC9 (16 isolates) and MRSA CC9 (16 isolates), and all of these isolates were tetracycline resistant and absent of IEC genes. These findings suggest that livestock-associated MRSA and MDRSA(CC9, IEC-negative, tetracycline-resistant) in humans is associated with occupational pig contact, not pet contact, and support growing concern about antibiotics use in pig farms and raising questions about the potential for occupational exposure to opportunistic *S. aureus*.

Modern food animal production is characterized by densely concentrated animals and routine antibiotic use, and this trend has increased globally. In the China in particular, the production of food animals has intensified with a shift towards larger operation, and it is extremely common to use non-therapeutic antimicrobials in feed to prevent animal disease, speed up animal growth and increase the efficiency of digestion rather than to treat bacterial infections^1^. There is increasing evidence that routine, non-therapeutic uses of antimicrobials in food animals increase the risk of propagation of multidrug-resistant bacteria, and recent studies also have shown that multidrug-resistant zoonotic bacteria can directly and indirectly colonize or infect humans via multiple environmental pathways^2^,^3^.

Concerns have been rising about the exchange of clonal complexes (CCs) of *S. aureus* between animals and humans^4^,^5^,^6^. Livestock-associated *S. aureus*, including multidrug-resistant *S. aureus* (MDRSA) and methicillin-resistant *S. aureus* (MRSA), has the capacity to colonize multiple livestock animals (including pigs, poultry, cows, and sheep), may facilitate colonization in people with animal contact, and can cause an array of severe infections in humans^5^,^6^. Nasal carriage and infections of livestock-associated *S. aureus* have been reported in Europe, the USA, and Asia^7^,^8^,^9^,^10^,^11^,^12^. Of particular interest is *S. aureus* CC398, which has been referred to as the most prevalent livestock-associated clone in European countries and North America^8^,^9^,^10^. It is notable that *S. aureus* CC9 is the most pandemic livestock-associated clone in most Asian countries, such as Taiwan, Hong Kong, and Thailand^7^,^11^,^12^. Despite a well-developed knowledge base of livestock-associated *S. aureus* in Asia^7^,^8^, little is known about methicillin- and multidrug-resistant *S. aureus* carriage among livestock workers and non-livestock workers in China.

It is noteworthy that few studies examining human *S. aureus* carriage have attempted to differentiate human- from livestock-associated isolates based on phenotypic and molecular markers. Studies have defined livestock-associated *S. aureus* based on CCs and resistance pattern^4^,^13^, while few have incorporated recent evidence suggesting that absence of the human-specific immune evasion cluster (IEC) genes (such as *scn*, *chp*, *sak*, and *sea*) may be useful markers of livestock association. Recent studies show that animal isolates are significantly less likely to carry IEC gene *scn* and absence of this molecular marker may aid in the differentiation of animal origins of *S. aureus* carried by livestock workers^9^,^14^,^15^. Thus, the IEC genes may be useful to differentiate livestock-to-human transmission from community-acquired human colonization.

Therefore, we undertook a cross-sectional study of pig workers, pet-owning workers, and control workers. The objective of our study was to compare the phenotypic and molecular characteristics of *S. aureus* isolates (including MDRSA and MRSA) carried by three participant groups, so as to reveal the association of occupational pig contact and pet contact with the transmission of animal-associated CCs. Additionally, we aimed to build on previous literature to examine a possible frequency-risk relation between occupational pig contact and human carriage of MDRSA and MRSA isolates.

## Results

### Participant characteristics

A total of 1,422 out of 1,444 interviewed individuals participated in this survey. Of them, 244 occupationally contacted with pigs (defined in this study as the pig workers, including 224 farm workers and 20 veterinarian doctors), 200 contacted with pets (the pet-owning workers), and the left 978 did not have any history of animal contact (the control workers). Pig workers were more males than pet-owning workers (p < 0.001) or controls (p < 0.001) (Table 1), and controls were older than pig workers(p < 0.001) and pet-owning workers (p < 0.001). There were also statistically significant differences among groups with regard to antibiotic use and medical facility visit in the last month (Table 1). This discrepancy was adjusted by applying the multivariable models.

### Prevalence of *S. aureus*, nonsusceptible *S. aureus*, MDRSA, and MRSA

As presented in Table 1, the overall prevalence of *S. aureus* nasal carriage among the study population was 10.3% (146/1,422) and was higher among pig workers than controls (15.2% vs. 8.8%; p = 0.003). The prevalence of nonsusceptible *S. aureus* was also higher among pig workers than controls (15.2% vs. 8.2%; p = 0.001). Notably, the prevalence of multidrug-resistant isolates was significant higher among pig workers than pet-owning workers (13.5% vs. 3.0%, p < 0.001, for MDRSA; 10.7% vs. 1.5%, p < 0.001, for MRSA) or controls (13.5% vs. 3.5%, p < 0.001, for MDRSA; 10.7% vs. 1.3%, p < 0.001, for MRSA).

### Group differences in *S. aureus* antibiotic resistance

**Figure 1:**
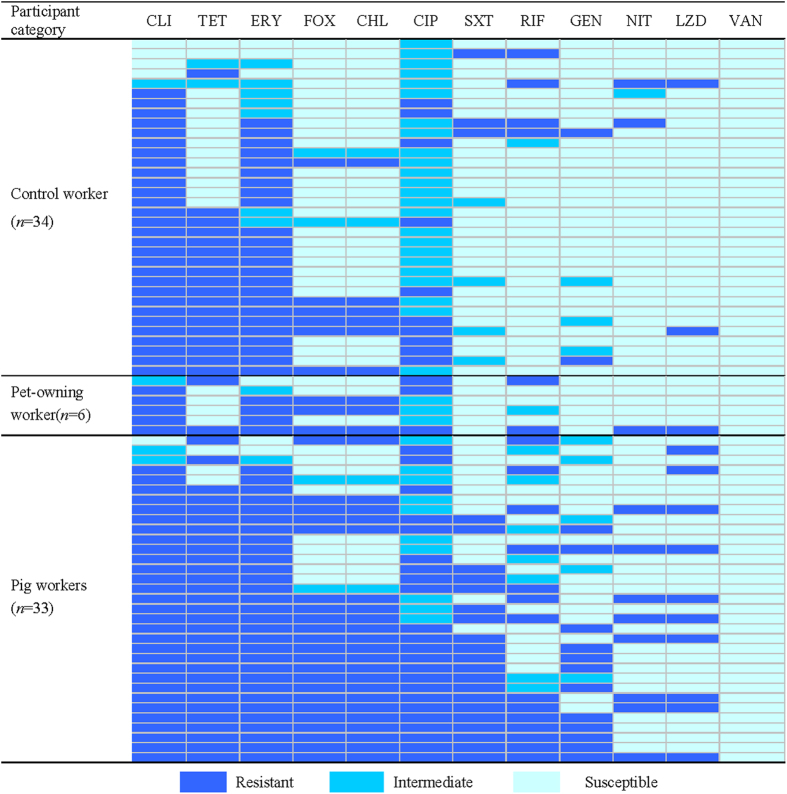
Heat map showing antibiotic resistance profiles of all multidrug-resistant *S. aureus* isolates from pig workers, pet-owning workers and control workers. Each row represents one isolate tested for susceptibility from a multidrug-resistant *S. aureus*-positive participant. (FOX, cefoxitin; CLI, clindamycin; ERY, erythromycin; TET, tetracycline; CHL, chloramphenicol; CIP, ciprofloxacin; RIF, rifampin; GEN, gentamicin; SXT, trimethoprim-sulfamethoxazole; NIT, nitrofurantoin; LZD, linezolid; VAN, vancomycin).

Susceptibility testing revealed that all *S. aureus* isolates were susceptible to vancomycin. Penicillin resistance was the most common phenotype observed in all groups, and most of the *S. aureus* strains from pig workers were nonsusceptible to clindamycin (91.9%; 34/37), tetracycline (89.2%; 33/37) and erythromycin (83.8%; 31/37). The dominant multidrug resistance pattern was nonsusceptible to clindamycin, erythromycin and tetracycline, with 78.4% (29/37) for pig workers, 20.9% (18/86) for controls, and 4.3% (1/23) for pet-owning workers (Fig. 1).

### Relations of pig contact with MDRSA and MRSA carriage

The prevalence of MDRSA carriage in pig workers was significantly higher than in controls [prevalence ratio (PR) = 3.38; 95% CI: 2.07–5.53], but the prevalence of MDRSA carriage in pet-owning workers was comparable to controls (PR = 0.73; 95% CI: 0.31–1.73) (Table 2). Similarly, we observed that the prevalence of MRSA carriage in pig workers was significantly higher than in controls (PR = 7.42; 95% CI: 3.71–14.83).

Compared with no occupational contact (including pet-owning workers and controls), pig workers experienced a higher prevalence of MDRSA carriage (PR = 3.57, 95%CI 2.22–5.74) and MRSA carriage (PR = 7.53, 95%CI 3.93–14.42). Frequency of pig contact (expressed a continuous variable) was associated with increased prevalence of MDRSA carriage (OR = 1.58, 95%CI 1.33–1.86) and MRSA carriage (OR = 2.06, 95%CI 1.63–2.59). Similarly, frequency of pig contact expressed as a categorized variable was also associated with prevalence of MDRSA carriage (OR = 1 for no contact; OR = 2.51 for < 8 hours per day; OR = 4.41 for ≥8 hours per day; p < 0.001 for linear trend) and MRSA carriage (OR = 1 for no contact; OR = 5.11 for <8 hours per day; OR = 9.54 for ≥8 hours per day; p < 0.001 for linear trend) in an increasing trend.

### Group differences in MLST typing of *S. aureus*

**Figure 2:**
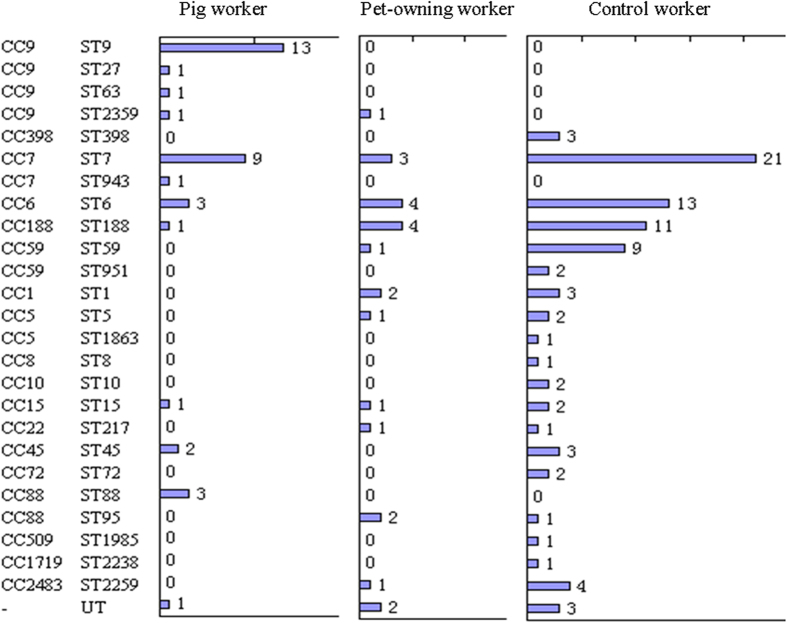
Sequence type diversity and distribution of *S. aureus* isolates from pig workers, pet-owning workers and control workers. Each bar represents the number of *S. aureus* isolates for each sequence types. (CC, clonal complex; ST, sequence type; NT, nontypeable).

MLST revealed a diversity of CCs, or clusters of closely-related sequence types, among participants with *S. aureus* (Fig. 2). Notably, the *S. aureus* CC9 (including ST9, ST27, ST63 and ST2359) was common among pig workers, and in addition MDRSA CC9 were only observed among pig workers and were absent among pet-owning workers and controls. ST7 and ST6 were found in all three participant groups. ST188 was common among *S. aureus* isolates from pet-owning workers and controls (17.4% and 12.8%, respectively), and ST59 was only common among isolates from controls (9 isolates).

### Group differences in markers of livestock association

As to the phenotypic marker of livestock association (tetracycline resistance), we observed that the proportion of tetracycline-resistant *S. aureus* carriage was significantly higher in pig workers than pet-owning workers (89.2% vs. 26.1%, p < 0.001) or controls (89.2% vs. 33.7%, p < 0.001), and tetracycline-resistant MRSA carriage was also significantly higher in pig workers than the other two groups (Table 3). Among methicillin-sensitive *S. aureus* (MSSA) isolates, tetracycline-resistant MSSA isolates were more prevalent among pig workers (81.8%) than pet-owning workers (20.0%) (p < 0.001) or controls (31.5%) (p < 0.001). As to the molecular markers (CC9 and IEC genes), pig workers demonstrated a greater proportion of *S. aureus* CC9, *scn*-negative *S. aureus* and *sak*-negative *S. aureus* carriage as compared with pet-owning workers and controls. Additionally, pig workers also demonstrated a greater proportion of MRSA CC9, *scn*-negative MRSA, *chp*-negative MRSA, *sak*-negative MRSA and *sea*-negative MRSA carriage as compared with pet-owning workers and controls.

Overlap between phenotypic and molecular markers of livestock association was predominantly observed among pig workers (Table 4). Notably, all 16 *S. aureus* CC9 among pig workers were MRSA isolates, and also exhibited other characteristics of livestock association (resistance to tetracycline and absence of the IEC genes *scn, chp, sak and sea*). While the single *S. aureus* CC9 (ST2359) observed among pet-owning workers was susceptible to methicillin and tetracycline, and carried the IEC genes *scn*, *chp* and *sak*, no overlap in these characteristics of livestock association was observed. Interestingly, three methicillin-susceptible isolates, all from controls, were identified as ST398, including two isolates absent of all four IEC genes and one isolate resistant to tetracycline.

## Discussion

This report investigates the livestock-associated strains of *S. aureus* CC9 (including MDRSA CC9 and MRSA CC9) based on phenotypic and molecular markers in Asia. The most striking finding from this study was that pig workers carried MRSA and MDRSA isolates with multiple markers of livestock-association (tetracycline-resistance, IEC-negative, and CC9), while pet-owning workers and controls were not observed to be carrying these livestock-associated isolates. Pig workers had a significantly higher prevalence of both MDRSA and MRSA than controls, but the prevalence of MDRSA and MRSA was similar in pet-owning workers and controls. Frequency of occupational pig contact was associated with increased prevalence of MDRSA and MRSA carriage in an increasing trend.

It is noteworthy that few studies examining carriage of *S. aureus* among livestock workers have attempted to differentiate human- from livestock-associated strains based on phenotypic and molecular markers. The *S. aureus* CC9, which has been identified as the most epidemic livestock-associated clone in most Asian countries^7^,^8^, is commonly considered as a molecular marker of livestock-association. Near universal resistance to tetracycline has been reported among *S. aureus* CC9 isolates from livestock and related workers^16^,^17^, and livestock-associated tetracycline-susceptible *S. aureus* also has been reported among humans absent of known livestock contact^13^,^18^. Our decision to purposively include pig workers, pet-owning workers and controls was to point out that the three groups differ in animal contact, use of antibiotics and sources of genetic stock. The prevalence of tetracycline-resistant *S. aureus* (including MRSA and MSSA) was relatively low among pet-owning workers or controls, but high among pig workers. Additionally, sixteen MRSA CC9 strains, all from pig workers, are uniformly resistant to tetracycline, suggesting that routine antibiotic use in livestock farms is an important cause for emergence of livestock-associated MRSA. Additionally, the recent study of historical isolates indicated that the tetracycline resistance gene *tet*(M) was present in 99% of the livestock-associated isolates but absent from the human-associated isolates^4^. Although tetracycline resistance is important to note with regard to its use in animal production, it is also used in plant culture and all kinds of human food sources^19^,^20^.

Recent evidence has revealed that the bacteriophage-encoded IEC genes are associated with human specificity, and absence of these genes may be a useful indicator of *S. aureus* CC398 livestock adaptation^9^,^14^,^15^. The study of MRSA CC398 originating from pigs versus humans indicated that human specificity genes (*chp*, *sak*, and *scn*) were found in human but not pig isolates^21^. Loss of the *scn* gene also has been observed during two independent human-to-animal host jumps by *S. aureus* CC398 and CC5, suggesting that the *scn* gene is selected against in animal hosts^4^,^22^. Of concern is that the relation between absence of IEC genes and *S. aureus* CC9 livestock adaptation is still unclear, so this study builds on previous literature to examine the potential association. In the present study, pig workers demonstrated a greater proportion of IEC-negative MRSA (including *scn*-negative, *chp*-negative, *sea*-negative, and *sak*-negative) as compared with pet-owning workers or controls. Interestingly, 16 MRSA CC9 from pig workers all exhibited the characteristics of resistance to tetracycline and absence of the IEC genes *scn, chp, sak and sea*, while the single methicillin-susceptible *S. aureus* CC9 (ST2359) observed among pet-owning workers was susceptible to tetracycline and carried the IEC genes *scn*, *chp* and *sak*, indicating substantial overlap in these characteristics of livestock association observed only among pig workers. None of the previous studies evaluated the relation of absence of IEC genes and *S. aureus* CC9 livestock adaptation, so we were unable to compare this result with others.

Previous studies have reported the potential for transmission of *S. aureus* between animals and humans on livestock operation sectors^9^,^23^,^24^,^25^. Studies conducted on livestock production sectors in Belgium and dairy farms in Dutch observed indistinguishable MRSA CC398 isolates in animals and related humans, suggesting an potential interspecies exchange of the same MRSA CC398^23^,^24^. In addition, most human MRSA isolates in Asian reports were the same as the MRSA isolates from pigs, namely ST9-t4358 in Malaysia, ST9-t899 in China and ST9-t899 in Taiwan, suggesting the possibility of interspecies exchange of the same MRSA CC9^11^,^16^,^17^. In this study, the findings revealed the pig workers demonstrated a higher prevalence of MDRSA and MRSA carriage as compared with pet-owning workers and controls, and also observed a positive relation between frequency of occupational pig contact and the risk of MDRSA or MRSA carriage as compared workers without occupational contact. Additionally, an interesting result in our study was that MRSA CC9 and MDRSA CC9 isolates were not recovered from pet-owning workers and controls, but only observed among pig workers, suggesting the probability of spread via direct and frequent livestock contact.

It is noteworthy that cases continue to be reported with no direct livestock-associated risk factors^11^,^26^,^27^. Persons living in areas of high livestock density were found to have a greater likelihood of livestock-associated MRSA carriage even if they lacked direct contact with animals^26^,^27^. A recent study from Taiwan reported that one of 16 regular visitors was found to colonize MRSA ST9^11^, as demonstrated for MRSA ST398 in Europe^28^,^29^, suggesting the probability of ST9 and ST398 transmission via human contact instead of animal contact. In this study, we observed two ST398 isolates with markers of livestock association (absent of all four IEC genes, tetracycline resistance), but all were from controls without animal contact in home and in workplaces. Some possible modes of exposure may involve person-to-person contact, contact with contaminated meat and environmental pathways such as air or waste releases from farms to the surrounding community. So the livestock-associated MRSA may pose an unpredictable future risk to humans. Future research should assess these factors in terms of their relations to the risk of human livestock-associated MRSA carriage.

To date, there have been only few reports on the prevalence and the risk factors of *S. aureus* and MRSA nasal carriage in China. This study indicated 8.8% to 15.2% *S. aureus* nasal carriage in Chinese workers from different groups, of which 1.3% to 10.7% were MRSA. Previous studies revealed 15.4% to 23.1% *S. aureus* nasal carriage in Chinese medical students from different regions, of which 3.0% to 9.4% were MRSA^30^,^31^. Another study revealed a similar nasal carriage rate (16.5%) in healthy people in Northern China with 8(0.33%) MRSA strains identified^32^. *S. aureus* nasal carriage in healthy adult populations has previously been suggested to be about 20–30% in European countries and in the United States^33^,^34^. In the study of nine European countries^34^, a wide range in nasal *S. aureus* carriage was also noted, with participants in Sweden having a prevalence more than double that of Hungarian participants (29.4% vs. 12.1%). Genetic factors have been reported to contribute to the *S. aureus* colonisation of an individual^35^, but these factors were not measured in most studies. Apart from geological and environmental restriction, the *S. aureus* prevalence can be influenced by the sampling site (only the anterior nares or multiple sites including the anterior nares, pharynx, skin, and perineum), cultivation (enrichment or not) and study design (cross-sectional study with once sampling or cohort study with repeated sampling). In view of the extent of the intercountry variations, future studies of *S. aureus* nasal carriage might explore reasons for these differences.

The advantage of this study was that we contributed additionally to the literature by differentiate human-from livestock-associated MDRSA and MRSA based on phenotypic and molecular markers. Additionally, we also examine the potential positive relation of frequency of occupational pig contact with human MDRSA and MRSA carriage. However, potential limitations also needed to be considered. Firstly, the study design is cross-sectional design, so we can only describe associations between livestock contact and MRSA carriage, not a causal conclusion. Results from this study need to be confirmed in a longitudinal study. A longitudinal design may also provide valuable information about dynamics and persistence of MRSA carriage in humans. Secondly, the results of this study do not suggest that pet contact plays a role in the transmission process of MDRSA or MRSA. Whether this finding is due to a lack of power, lack of colonization of companion animals, or lack of transmission cannot be determined. Previous studies have indicated that pets were acting as potential reservoirs for human infection of MRSA in the community^36^, whereas a recent review concluded that available data on MRSA transmission between pets and humans are limited and that the public health impact of such transmission needs to be the subject of more detailed epidemiological studies^37^.

In conclusion, our findings indicate livestock-associated MRSA and MDRSA (CC9, IEC-negative, tetracycline-resistant) in humans is associated with occupational pig contact, not pet contact, suggesting a potential for livestock-to-human transmission of *S. aureus* by occupational livestock contact. These findings support growing concern about modern food-animal production characterized by densely concentrated animals and routine antibiotic use, and raise questions about the potential for occupational exposure to opportunistic and multidrug-resistant isolates of *S. aureus* which have become leading causes of nosocomial and community-acquired infections in China and in countries worldwide.

## Materials and Methods

### Ethics statement

The study was approved by the ethics committee of Guangdong Pharmaceutical University, and was carried out in accordance with the approved guidelines. Before participating, all participants signed an informed consent form.

### Study design and recruitment

A cross-sectional study was conducted between November 2013 and November 2014 in Guangdong province, China by researchers from the Guangdong Pharmaceutical University. A two-stage sample design was used to obtain a representative sample. Firstly, four cities were randomly sampled from 21 cities in Guangdong province. Secondly, in each city, we selected a specific number of pig farms to reach a respondent sample size of 60 workers with occupational pig contact and at the same time selected two factories to reach a sample size of 300 nonfarm workers (i.e., workers from the hardware factory or the biscuit factory, including pet-owning workers and control workers without animal contact). Through this study, we enrolled three categories of participants, including pig workers, pet-owning workers, and control workers.

The eligibility criteria for workers included: (1) being 15 years or older, (2) being able to speak and understand Chinese, (3) not working at a health care facility, and (4) without occupational livestock contacts for control workers. In each of the selected farms or factories, we asked the chief to provide a list of staff meeting the above criteria. All workers on the list were approached by our research team to participate in the survey through face-to-face interviews using questionnaires.

### Isolation and identification of *S. aureus* and MRSA

After completing the questionnaire, study personnel obtained a nasal swab from both nares of each participant. Swabs were soaked in 2 ml of enrichment broth containing 1% tryptone, 1% mannitol, 7.5% NaCl and 0.25% yeast extract, and incubated overnight at 35 ± 1 °C. To isolate *S. aureus*, a loopful of the broth was streaked onto mannitol salt agar and incubated at 37 °C for 24 hours. Presumptive colonies were streaked to 5% sheep blood agar plates and incubated at 35 °C overnight. Repeated subcultivation of specimen was performed as needed if the incubations were mixed with multiple unrecognized colonies. Initial identification of *S. aureus* was based on gram staining, morphology and traditional biochemical tests, including catalase, DNase and tube coagulase tests. We also performed polymerase chain reaction (PCR) assays targeting *S. aureus* 16S rRNA, *nuc* and *mecA* genes^38^.

### Antibiotic susceptibility testing

The antibiotic susceptibility profiles (penicillin, cefoxitin, clindamycin, tetracycline, erythromycin, chloramphenicol, ciprofloxacin, rifampin, gentamicin, trimethoprim-sulfamethoxazole, nitrofurantoin and linezolid) were assessed by the Kirby-Bauer disk diffusion method according to Clinical and Laboratory Standards Institute (CLSI) guidelines^39^. Vancomycin susceptibility was determined by minimum inhibitory concentration (MIC) test onto Mueller-Hinton agar and vancomycin agar screen test onto brain heart infusion agar^39^,^40^. *S. aureus* strain ATCC 25923 was used as a control strain. According to CLSI guidelines, we classified the isolates as susceptible, intermediate, or resistant to each antibiotic. In addition, we also classified the isolates as either nonsusceptible (including both intermediate and resistant isolates) or susceptible. Cefoxitin-resistant isolates were identified as MRSA. *S. aureus* isolates were classified as MDRSA if they were nonsusceptible to ≥3 classes of antibiotics or were MRSA^41^,^42^.

### Molecular testing

Multilocus sequence typing (MLST) of the seven housekeeping genes was performed and analyzed as previously described^37^. *S. aureus* isolates were assigned to standardized sequence types using the open-access MLST database (http://www.mlst.net/). The presence of IEC genes (including *scn*, *chp*, *sak* and *sea*) were determined by a PCR strategy described previously^43^.

### Data analysis

STATA version 13.0 (StataCorp LP, College Station, Texas, USA) was utilized for statistical analysis. A two-sided p-value ≤ 0.05 was considered statistically significant. To compare qualitative and quantitative data between groups, we used Analysis of Variance (ANOVA), chi-square test, and post hock test with Bonferroni adjustment. The relation between occupational pig contact and carriage of MDRSA or MRSA isolates was examined using univariable and multivariable log binomial regression models. Linear trends of pig contact were assessed by modeling frequency of pig contact (hours per day) as continuous variables (logarithmic scale) or categorized variables (no contact, <8 hours per day, or ≥8 hours per day) in log binomial models. Based on *a priori* assumptions, all multivariable models were adjusted for sex, age groups(15–24, 25–34,35–44, and 45–60 years), self-reported use of antibiotics in the last month (yes or no), and any self-reported visit to a medical facility (including clinics, hospitals, community health station, and nursing homes) in the last month (yes or no).

## Additional Information

**How to cite this article**: Ye, X. *et al.* Livestock-associated methicillin and multidrug resistant *S. aureus* in humans is associated with occupational pig contact, not pet contact. *Sci. Rep.* **6**, 19184; doi: 10.1038/srep19184 (2016).

## Figures and Tables

**Table 1:** Study population characteristics by participant category, in Guangdong, China.

Characteristics	Total	Pig worker	Pet-owning worker	Control worker	*p-*Value			
					*p*	*p*_1_	*p*_2_	*p*_3_
Age(years)								
15–24	176(12.4)	38(15.6)	32(16.0)	106(10.8)	<0.001	<0.001	<0.001	<0.001
25–34	429(30.1)	124(50.8)	69(34.5)	236(24.1)				
35–44	483(34.0)	43(17.6)	68(34.0)	372(38.0)				
45–60	334(23.5)	39(16.0)	31(15.5)	264(27.0)				
Sex, male	841(59.1)	188(77.1)	85(42.5)	568(58.1)	<0.001	<0.001	<0.001	<0.001
Antibiotic use in the last month	694(48.9)	154(63.1)	103(51.8)	437(44.7)	<0.001	0.016	<0.001	0.070
Medical facility visit in the last month	580(40.8)	111(45.5)	101(50.5)	368(37.6)	0.001	0.293	0.024	0.001
Prevalence								
*S. aureus*	146(10.3)	37(15.2)	23(11.5)	86(8.8)	0.011	0.261	0.003	0.229
Nonsusceptible *S. aureus*^a^	139(9.8)	37(15.2)	22(11.0)	80(8.2)	0.004	0.198	0.001	0.196
MDRSA^b^	73(5.1)	33(13.5)	6(3.0)	34(3.5)	<0.001	<0.001	<0.001	0.735
MRSA	42(3.0)	26(10.7)	3(1.5)	13(1.3)	<0.001	<0.001	<0.001	0.849

Note. Values are expressed as number of participants (the proportion of participants surveyed), except where specified otherwise.

MDRSA, multidrug-resistant *S. aureus*; MRSA, methicillin-resistant *S. aureus*.

*p*: Comparison among pig workers, pet-owning workers, and control workers.

*p*_1_: Comparison between pig workers and pet-owning workers.

*p*_2_: Comparison between pig workers and control workers.

*p*_3_: Comparison between pet-owning workers and control workers.

^a^*S. aureus* intermediate or resistant to any antibiotic class.

^b^Including *S. aureus* isolates nonsusceptible to ≥3 classes of antibiotics or MRSA isolates.

**Table 2:** Estimates of the association of exposures with MDRSA carriage and MRSA carriage, in Guangdong, China.

Category	*n*	Carriage of MDRSA		Carriage of MRSA	
		Unadjusted PR (95% CI)	Adjusted PR (95% CI)^a^	Unadjusted PR(95%CI)	Adjusted PR(95% CI)^a^
Participant group					
Control worker	978	Referent	Referent	Referent	Referent
Pet-owning worker	200	0.86(0.37–2.03)	0.73(0.31–1.73)	1.13(0.32–3.92)	0.93(0.26–3.25)
Pig worker	244	3.89(2.46–6.14)	3.38(2.07–5.53)	8.01(4.18–15.37)	7.42(3.71–14.83)
With occupational pig contact					
No^b^	1178	Referent	Referent	Referent	Referent
Yes	244	3.98(2.57–6.18)	3.57(2.22–5.74)	7.85(4.27–14.40)	7.53(3.93–14.42)
Frequency of pig occupational contact(hours per day)					
No	1178	Referent	Referent	Referent	Referent
<8	94	3.13(1.62–6.06)	2.51(1.26–4.98)	6.27(2.75–14.26)	5.11(2.16–12.09)
≥8	150	4.52(2.78–7.33)	4.41(2.62–7.41)	8.84(4.60–16.95)	9.54(4.77–19.06)
Frequency of occupational pig contact(hours per day)^c^		1.63(1.40–1.91)	1.58 (1.33–1.86)	2.08(1.68–2.57)	2.06(1.63–2.59)

Note. *n*, number of participants; MDRSA, multidrug-resistant *S. aureus*; MRSA, methicillin-resistant *S. aureus*; PR, prevalence ratio; CI, confidence interval.

^a^Adjusted for sex, age, antibiotic use in the last month, and medical facility visit in the last month.

^b^Use pet-owning workers and control workers as no occupational pig contact.

^c^Use logarithmic exposure in the model.

**Table 3:** Phenotypic and molecular characteristics of *S. aureus* carriage among study participants, in Guangdong, China.

Characteristics	Total (*n* = 146)	Pig worker (*n* = 37)	Pet-owning worker(*n* = 23)	Control worker [*n* = 86(%)]	*p-*Value			
					*p*	*p*_1_	p_2_	p_3_
Tetracycline-resistant *S.aureus*	68(46.6)	33(89.2)	6(26.1)	29(33.7)	<0.001	<0.001	<0.001	0.486
Tetracycline-resistant MRSA	32(21.9)	24(64.9)	2(8.7)	6(7.0)	<0.001	<0.001	<0.001	0.779
*S. aureus* CC9	17(11.6)	16(43.2)	1(4.4)	0(0.0)	<0.001	0.001	<0.001	0.052
MRSA CC9	16(11.0)	16(43.2)	0(0.0)	0(0.0)	<0.001	<0.001	<0.001	—
*scn*-negative *S. aureus*	64(43.8)	26(70.3)	5(21.7)	33(38.4)	<0.001	<0.001	0.001	0.137
*scn*-negative MRSA	31(21.2)	23(62.2)	1(4.4)	7(8.1)	<0.001	<0.001	<0.001	0.536
*chp*-negative *S. aureus*	105(71.9)	31(83.8)	16(69.6)	58(67.4)	0.174	0.194	0.063	0.846
*chp*-negative MRSA	34(23.3)	23(62.2)	2(8.7)	9(10.5)	<0.001	<0.001	<0.001	0.802
*sak*-negative *S. aureus*	66(45.2)	26(70.3)	5(21.7)	35(40.7)	<0.001	<0.001	0.003	0.094
*sak*-negative MRSA	31(21.2)	24(64.9)	1(4.4)	6(7.0)	<0.001	<0.001	<0.001	0.648
*sea*-negative *S. aureus*	130(89.0)	35(94.6)	17(73.9)	78(90.7)	0.033	0.022	0.498	0.033
*sea*-negative MRSA	39(26.7)	25(67.6)	2(8.7)	12(14.0)	<0.001	<0.001	<0.001	0.503

Note. Values are expressed as number of *S. aureus* isolates (the proportion of *S. aureus* isolates), except where specified otherwise.

MRSA, methicillin-resistant *S. aureus*.

*p*: Comparison among pig workers, pet-owning workers, and control workers.

*p*_1_: Comparison between pig workers and pet-owning workers.

*p*_2_: Comparison between pig workers and control workers.

*p*_3_: Comparison between pet-owning workers and control workers.

-: No estimate of *p v*alue is provided due to no occurrence of the outcome of interest in the two groups.

**Table 4:** Phenotypic and molecular characteristics of livestock-associated *S.aureus* carried by study participants, in Guangdong, China.

Participant category	CC	MLST	*scn*	*chp*	*sak*	*sea*	tetracycline	methicillin
Pig workers (n = 16)	CC9	ST9	−	−	−	−	R	R
	CC9	ST9	−	−	−	−	R	R
	CC9	ST9	−	−	−	−	R	R
	CC9	ST9	−	−	−	−−	R	R
	CC9	ST9	−	−	−	−	R	R
	CC9	ST9	−	−	−	−	R	R
	CC9	ST9	−	−	−	−	R	R
	CC9	ST9	−	−	−	−	R	R
	CC9	ST9	−	−	−	−	R	R
	CC9	ST9	−	−	−	−	R	R
	CC9	ST9	−	−	−	−	R	R
	CC9	ST9	−	−	−	−	R	R
	CC9	ST9	−	−	−	−	R	R
	CC9	ST27	−	−	−	−	R	R
	CC9	ST63	−	−	−	−	R	R
	CC9	ST2359	−	−	−	−	R	R
Pet-owning worker(n = 1)	CC9	ST2359	+	+	+	−	S	S
Control worker (n = 3)	CC398	ST398	−	−	−	−	S	S
	CC398	ST398	−	−	−	−	R	S
	CC398	ST398	+	+	+	−	S	S

CC, clonal complex; ST, sequence type; +, positive; −, negative; R, resistant; S, susceptible.
